# Diagnostic and therapeutic impact of implantable cardiac monitors across different clinical indications: a real-world cohort

**DOI:** 10.1093/ehjopen/oeag028

**Published:** 2026-02-17

**Authors:** Margarida Castro, Lucy Calvo, Luísa Pinheiro, Mariana Tinoco, Bárbara Lage Garcia, Ana Cristina Paredes, Assunção Alves, Bernardete Rodrigues, João Português, Sílvia Ribeiro, Victor Sanfins, António Lourenço

**Affiliations:** Cardiology Department, Unidade Local de Saúde do Alto Ave, Rua dos Cutileiros, Creixomil, Guimarães 4835-044, Portugal; Cardiology Department, Unidade Local de Saúde do Alto Ave, Rua dos Cutileiros, Creixomil, Guimarães 4835-044, Portugal; Cardiology Department, Unidade Local de Saúde do Alto Ave, Rua dos Cutileiros, Creixomil, Guimarães 4835-044, Portugal; Cardiology Department, Unidade Local de Saúde do Alto Ave, Rua dos Cutileiros, Creixomil, Guimarães 4835-044, Portugal; Cardiology Department, Unidade Local de Saúde do Alto Ave, Rua dos Cutileiros, Creixomil, Guimarães 4835-044, Portugal; Association P5 Digital Medical Center (ACMP5), Campus de Gualtar Escola de Medicina da Universidade do Minho, Braga 4710-057, Portugal; Cardiology Department, Unidade Local de Saúde do Alto Ave, Rua dos Cutileiros, Creixomil, Guimarães 4835-044, Portugal; Cardiology Department, Unidade Local de Saúde do Alto Ave, Rua dos Cutileiros, Creixomil, Guimarães 4835-044, Portugal; Cardiology Department, Unidade Local de Saúde do Alto Ave, Rua dos Cutileiros, Creixomil, Guimarães 4835-044, Portugal; Cardiology Department, Unidade Local de Saúde do Alto Ave, Rua dos Cutileiros, Creixomil, Guimarães 4835-044, Portugal; Cardiology Department, Unidade Local de Saúde do Alto Ave, Rua dos Cutileiros, Creixomil, Guimarães 4835-044, Portugal; Cardiology Department, Unidade Local de Saúde do Alto Ave, Rua dos Cutileiros, Creixomil, Guimarães 4835-044, Portugal

**Keywords:** Implantable cardiac monitor, Atrial fibrillation, Syncope, ESUS

## Abstract

**Graphical Abstract:**
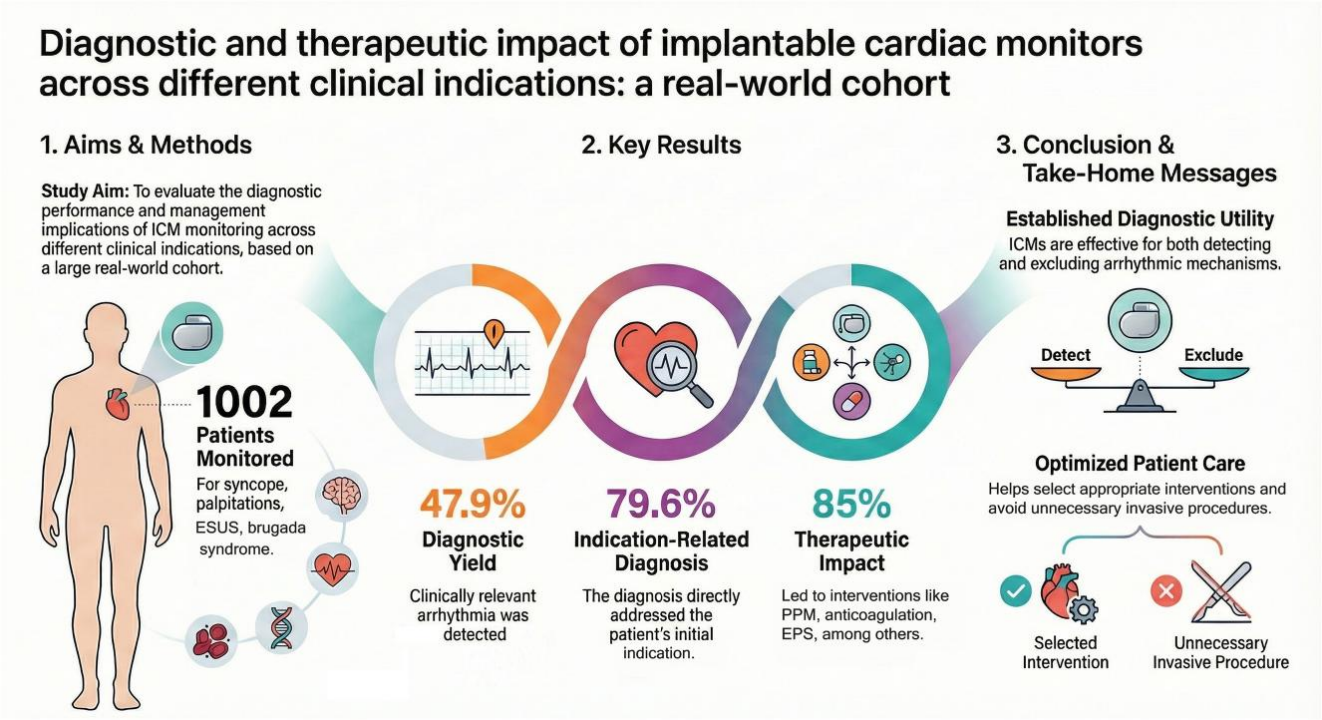

Implantable cardiac monitors (ICMs) are widely used for long-term rhythm monitoring in patients with suspected arrhythmic disorders.^1^ Although their diagnostic yield has been demonstrated across multiple clinical scenarios, less is known about how ICM-derived findings translate into therapeutic decision-making according to the initial indication for implantation.^2^

Despite guideline endorsement,^3^ given the costs and resource demands associated with prolonged continuous monitoring, a critical appraisal of the incremental value of ICM use in routine practice is warranted.

We retrospectively analysed 1002 consecutive patients undergoing ICM implantation at a tertiary referral centre between June 2004 and August 2024. Indications included unexplained syncope or presyncope (655, 65.4%), palpitations (149, 14.9%), embolic stroke of undetermined source (ESUS) (117, 11.7%), and arrhythmic risk stratification in Brugada syndrome (64, 6.4%). Device recordings and medical records were reviewed to assess diagnostic yield (proportion of ICMs yielding a diagnosis), time to diagnosis, indication-related diagnostic rate (IDR; proportion of diagnoses consistent with the indication), and therapeutic impact (proportion of diagnoses leading to a management change). Groups were compared using one-way ANOVA/Kruskal–Wallis, χ^2^, or Fisher’s exact test.

All patients underwent baseline evaluation including a 12-lead ECG, transthoracic echocardiogram, and at least 24-h electrocardiographic monitoring prior to implantation; additional investigations [including tilt-table testing and electrophysiology study (EPS)] were performed at the physician’s discretion according to clinical presentation. Importantly, patients with syncope and clear reflex features in whom tilt-table testing or other investigations provided a definitive diagnosis did not proceed to ICM implantation and were therefore not included in this cohort. Overall, prior to implantation, 24-h Holter monitoring was performed in 900 patients (89.8%), EPS in 115 (11.5%), and tilt-table testing in 70 (7.0%). Automatic alerts and patient-triggered recordings were analysed by trained technicians and confirmed by an experienced electrophysiologist.

*Table 1* summarizes patient characteristics and outcomes.

**Table 1:** Comparison of baseline characteristics and diagnostic outcomes across indication groups

Variable	Overall (*n* = 1002)^a^	Syncope/presyncope (*n* = 655)	Palpitations (*n* = 149)	ESUS (*n* = 117)	Brugada syndrome (*n* = 64)	Adj *p* (FDR)^c^	ES
Baseline characteristics							
Mean age at implant^d^, years (±SD)	65.71 ± 15.2	68.93 ± 14.16^f^	61.62 ± 15.94^g^	61.51 ± 12.28^g^	52.41 ± 15.02^h^	**<0.001**	0.10
Gender (female) *n* (%)	490 (48.9)	329 (50.2)	83 (55.7)	49 (41.9)	26 (40.6)	0.077	0.85
Underlying cardiac conditions							
Structural heart disease, *n* (%)	206 (20.6)	152 (23.3)^f^	33 (22.1)^f,g^	13 (11.1)^h^	2 (3.1)^g,h^	**<0.001**	0.15
Coronary artery disease, *n* (%)	115 (11.5)	86 (13.1)	13 (8.7)	11 (9.4)	2 (3.1)	0.05	0.09
Reduced EF, *n* (%)	85 (8.5)	61 (9.3)	14 (9.4)	6 (5.1)	1 (1.6)	0.091	0.08
Rhythm, *n* (%)							
Sinus	942 (94.0)	596 (91.0)	149 (100)	117 (100)	64 (100)		
AF/AFL	60 (6.0)	59 (9.0)	0	0	0		
Conduction disturbance, *n* (%) [1]	309 (30.8	244 (37.3)	24 (16.1)^g^	21 (17.9)^g^	13 (20.3)^g^	**<0.001**	0.20
First-degree AV block [2]	130 (13.0)	103 (15.7)^f^	7 (4.7)^g^	8 (6.8)^f,g^	6 (9.4)^f,g^	**<0.001**	0.14
Left anterior fascicular block [2]	116 (11.6)	100 (15.3)^f^	7 (4.7)^g^	4 (3.4)^g^	4 (6.3)^f,g^	**<0.001**	0.16
RBBB [2]	99 (9.9)	78 (11.9)^f^	7 (4.7)^f^	6 (5.1)^f^	8 (12.5)^f^	**0.013**	0.11
LBBB [2]	79 (7.9)	65 (9.9)^f^	7 (4.7)^f,g^	4 (3.4)^f,g^	0^g^	**<0.001**	0.12
Bifascicular block^e^ [1]	52 (5.2)	48 (7.3)^f^	1^g^ (0.7)	0^g^	3^f,g^ (4.7)	**<0.001**	0.14
Pre-implant investigation, *n* (%)							
Holter monitoring	900 (89.8)	581 (88.7)^f,g^	139 (93.3)^g,h^	114 (97.4)^h^	50 (78.1)^f^	**<0.001**	0.14
EPS	115 (11.5)	52 (7.9)^f,g^	20 (13.4)^g^	2 (1.7)^f^	36 (56.3)^h^	**<0.001**	0.39
Tilt test	70 (7.0)	60 (9.2)^f^	4 (2.7)^f,g^	0^g^	6 (9.3)^f^	**<0.001**	0.14
Diagnostic outcomes							
Diagnosis, *n* (diagnostic yield^f^, %)	480 (47.9)	331 (50.5)^f^	82 (55.0)^f^	41 (35.0)^g^	17 (26.6)^g^	**<0.001**	0.16
Bradyarrhythmia^b^	250 (52.1)	232 (70.1)^f^	4 (4.9)^g^	5 (12.2)^g^	2 (11.8)^g^	**<0.001**	0.57
Complete AVB	95 (19.8)	83 (25.1)	1 (2.0)	3 (7.3)	2 (11.8)		
SSS	143 (29.8)	139 (42.0)	2 (2.4)	2 (4.9)	0		
Tachyarrhythmia^b^	230 (47.9)	99 (29.9)^f^	78 (95.1)^g^	36 (87.8)^g^	15 (88.2)^g^		
AF/AFL	151 (31.5)	59 (17.8)	53 (64.6)	32 (78.0)	6 (35.3)		
SVT	43 (9.0)	18 (5.4)	16 (19.5)	3 (7.3)	7 (41.1)		
VT	23 (4.8)	15 (4.5)	4 (6.1)	0	1 (5.9)		
Symptomatic correlation with event, *n* (%)	262 (54.6)	201 (60.7)^f^	42 (51.2)^f^	7 (17.1)^g^	10 (58.8)^f^	**<0.001**	0.25
Indication-related diagnostic, *n* (IDR^g^, %)	382 (79.6)	255 (77.0)^f^	77 (93.9)^g^	32 (78.0)^f,g^	11 (64.7)^f^	**0.001**	0.17
Therapeutic impact, *n* (TIR^h^, %)	408 (85.0)	287 (86.7)	67 (81.7)	32 (78.0)	14 (82.4)	0.322	0.07
PPM implant	196 (48.0)	191 (66.6)	0	2 (6.3)	1 (7.1)		
Anticoagulation	119 (29.2)	45 (15.7)	38 (56.7)	30 (93.8)	5 (35.7)		
EPS	43 (10.5)	16 (5.6)	22 (38.7)	0	6 (42.9)		
ICD implant	21 (5.1)	15 (5.2)	3 (4.5)	0	1 (7.1)		
CRTD implant	4 (0.98)	4 (1.4)	0	0	0		
CRTP implant	3 (0.7)	3 (1.0)	0	0	0		
CPAP	8 (2.0)	5 (1.7)	1 (1.5)	1 (3.1)	1 (7.1)		
Cardioneuroablation	3 (0.7)	2 (0.7)	0	0	0		
ECV	3 (0.7)	1 (0.3)	2 (3.0)	0	0		
Others	6 (0.6)	5 (0.8)	1 (1.5)	0	0		
Time to indication-related diagnostic	(n = 354)	(n = 244)	(n = 65)	(n = 31)	(n = 9)		
Months (M ± SD)	12.30 (12.41)	11.65 ± 11.51	13.09 ± 14.49	15.89 ± 15.00	12.81 ± 11.76	0.322	0.010

Square brackets [*n*] indicate missing observations. Bold values are statistically significant at *P* ≤ 0.05. The values with different subscript letters in a row are significantly different (*P* ≤ 0.05) in *post hoc* comparisons (Tukey or Games–Howell).

AF, atrial fibrillation; AFL, atrial flutter; AV, atrioventricular; AVB, atrioventricular block; CPAP, continuous positive airway pressure; CRTD, cardiac resynchronization therapy defibrillator; CRTP, cardiac resynchronization therapy pacemaker; ECV, electrical cardioversion; EF, ejection fraction; EPS, electrophysiology study; ES, effect size; ESUS, embolic stroke of undetermined source; FDR, false discovery rate; ICD, implantable cardioverter–defibrillator; LBBB, left bundle branch block; MI, myocardial infarction; PM, pacemaker; RBBB, right bundle branch block; SSS, sick sinus syndrome; SVT, supraventricular tachycardia; VT, ventricular tachycardia.

^a^Seventeen participants (1.7%) were not included in these analyses due to the small sample size and heterogeneity of indications grouped as ‘Others’.

^b^Other diagnoses (e.g. symptomatic second-degree AVB) were omitted from the table for clarity and simplicity.

^c^ *P*-values adjusted for multiple comparisons using the Benjamini–Hochberg false discovery rate (FDR).

^d^Results for robust test of equality of means Welch’s test.

^e^Bifascicular block was defined as RBBB and left anterior/posterior fascicular block.

^f^Diagnostic yield: proportion of ICMs that resulted in a definitive rhythm diagnosis (number of diagnosis/number of implanted devices).

^g^IDR: proportion of diagnostic findings consistent with the original indication for ICM implantation, reflecting the alignment between pre-implantation clinical suspicion and final rhythm diagnosis (IDR, number of consistent diagnosis/total number of diagnosis).

^h^TIR: proportion of diagnoses that led to a therapeutic intervention or management change (TIR, therapeutic actions/number of diagnosis).

Over a median time to diagnosis of 8 months, the overall diagnostic yield was 47.9% (480/1002). Among patients with a diagnostic finding, results were concordant with the initial indication in 79.6% (382/480) and led to therapeutic intervention in 85.0% (408/480), with no significant differences across indication groups.

Diagnostic outcomes varied by indication: bradyarrhythmias predominated in syncope/presyncope (70.1%), frequently prompting permanent pacemaker (PPM) implantation (66.6%), whereas tachyarrhythmias were more commonly identified in patients investigated for palpitations (95.1%) or ESUS (87.8%), leading to antiarrhythmic therapy, anticoagulation, or EPS. Overall, among patients with an ICM-based diagnosis, 66.2% underwent an invasive procedure.

Arrhythmic diagnoses accrued predominantly within the first year of monitoring. By 6 months, 35.7% of tachyarrhythmic and 43.1% of bradyarrhythmic diagnoses had been established, increasing to 60.5% and 64.4%, respectively, at 1 year. Symptom–rhythm correlation was documented in 54.6% of patients with recorded events. Importantly, symptom recurrence without associated arrhythmia also occurred across indications (5.5% in syncope/presyncope, 4.0% in palpitations, and 11.0% in Brugada group), underscoring the clinical value of ICM monitoring not only for arrhythmia detection but also for exclusion of arrhythmic mechanisms, avoidance of unnecessary invasive procedures, and facilitation of alternative diagnostic pathways, including non-cardiac causes, such as reflex, neurological, or psychogenic syncope. The inclusion of presyncope within the syncope group may have diluted its diagnostic yield, as presyncopal episodes are often multifactorial.

Our findings confirm and extend the real-world data reported by Smith *et al.* and align with the recent large observational study by Letsas *et al.*, showing comparable diagnostic yield and therapeutic impact.^1,2^ Consistent with prior registry data, diagnostic yield was highest in patients implanted for palpitations or syncope/presyncope and lower in those monitored for ESUS or Brugada syndrome, supporting the concept that continuous rhythm monitoring is most effective when symptoms are frequent or directly related to rhythm disturbances.^4,5^ A valuable feature of this study was the assessment of indication-related diagnostic value, with an indication-related diagnostic rate of 79.6%, comparable to previous large registries.^2^ While bystander arrhythmias are an inherent limitation of prolonged monitoring, most diagnoses in our cohort were clinically concordant with the original indication and translated into therapeutic action. Most indication-related diagnoses occurred early after implantation, declining beyond 18 months of follow-up.^1,2,4,6–9^

In patients with Brugada syndrome, diagnostic yield was lower (26.6%), reflecting the unpredictable and infrequent nature of spontaneous arrhythmic events in this population. Nevertheless, ICM monitoring proved valuable both for detection of clinically relevant arrhythmias and for exclusion of arrhythmic causes of symptoms, thereby preventing unnecessary implantable cardioverter–defibrillator implantation in selected cases.

In this real-world cohort, extended external monitoring was not routinely performed prior to ICM implantation, reflecting contemporary guideline-based practice in patients with infrequent symptoms occurring at intervals longer than 30 days.^10^ Tilt-table testing and cardiovascular autonomic assessment remain important components of the diagnostic work-up of suspected reflex syncope and orthostatic intolerance. Importantly, patients with syncope and typical reflex features in whom autonomic assessment or other investigations were diagnostic generally did not proceed to ICM implantation and were therefore not captured in this analysis; thus, the observed rate of tilt-table testing should be interpreted in light of this selection process. Accordingly, ICM implantation was generally reserved for patients with infrequent events, atypical clinical features, or high-risk presentations, in whom symptom–rhythm correlation is essential. In the PICTURE registry,^4^ approximately one-third of patients with syncope underwent prior tilt-table testing; nevertheless, ILR monitoring guided the diagnosis in nearly 80% of patients with recurrent syncope, highlighting the incremental value of continuous rhythm monitoring beyond initial reflex syncope evaluation. Finally, the documentation of symptom recurrence without arrhythmia constituted clinically actionable information, enabling de-escalation of cardiac investigations and supporting alternative diagnostic pathways, including autonomic, neurological, or psychogenic mechanisms.

This retrospective single-centre study has inherent limitations, including potential referral and reporting biases, device-related detection constraints, and follow-up duration limited by battery longevity. Missing data inherent to the retrospective design may have affected some analyses; however pairwise deletion was applied to maximize data use and reduce bias. Nonetheless, these findings reflect contemporary real-world practice and highlight that ICMs function not only as diagnostic tools but also as instruments that shape clinical decision-making and management across different indications.

## Data Availability

The data used to support the findings of this study are available from the corresponding author upon request.

## Data availability

The data supporting the findings of this study are available within the article and its supplementary material.
